# Proton radiation impairs mechanotransduction in C2C12 myoblasts

**DOI:** 10.1038/s41526-026-00609-w

**Published:** 2026-05-14

**Authors:** Áron Gere, László Szabó, Brigitta Tillmann, Csaba Balogh, Florina Zákány, Tamás Kovács, Péter Nagy, Máté Szarka, László Csernoch, Beatrix Dienes

**Affiliations:** 1 Department of Physiology, Faculty of Medicine, University of Debrecen, Debrecen, Hungary; 2 Doctoral School of Molecular Medicine, Faculty of Medicine, University of Debrecen, Debrecen, Hungary; 3 HUN-REN DE Cell Physiology Research Group, University of Debrecen, Debrecen, Hungary; 4 Department of Biophysics and Cell Biology, Faculty of Medicine, University of Debrecen, Debrecen, Hungary; 5 HUN-REN Institute for Nuclear Research, Debrecen, Hungary; 6 Vitrolink Llc, Debrecen, Hungary

**Keywords:** Biophysics, Cell biology, Physiology

## Abstract

Skeletal muscle dysfunction during spaceflight arises from combined mechanical and non-gravitational stressors. Here, we modeled solar particle event-like proton irradiation in C2C12 myogenic cells to examine radiation-induced membrane and mechanosensitive channel alterations. Proton exposure increased membrane rigidity in a dose-dependent manner and significantly reduced the pharmacological activatability of Piezo1 channels, identifying membrane-mediated mechanotransduction as a potential contributor to radiation-induced muscle dysfunction.

## Background and rationale

As human space exploration transitions from the relatively protected environment of Low Earth Orbit (LEO) to long-duration missions targeting the Moon and Mars, understanding how human physiology adapts to the hostile conditions of deep space has become a mission-critical priority. Among the many physiological challenges, preservation of the musculoskeletal system is paramount. Skeletal muscle is exceptionally plastic yet highly susceptible to environmental stressors, exhibiting rapid and profound maladaptation in the absence of gravitational loading^1,2^.

Skeletal muscle function depends not only on biochemical cues but also on the continuous integration of mechanical signals required to maintain contractile performance, structural integrity, and regenerative capacity. Consequently, disruption of mechanosensitive signaling pathways represents a critical vulnerability under extreme environmental conditions such as spaceflight. Muscle atrophy and weakness during spaceflight have traditionally been attributed to microgravity-induced unloading, which suppresses protein synthesis through Akt/mTOR signaling, enhances proteolysis via FOXO pathways, and alters mitochondrial and oxidative homeostasis involving AMPK–ULK1 signaling^3–5^. However, increasing evidence suggests that impaired muscle regeneration and altered mechanotransduction may not solely represent downstream consequences of microgravity-induced unloading, but may also be directly affected by additional space-related stressors, thereby contributing to muscle dysfunction^6,7^.

In addition to microgravity, ionizing radiation constitutes a major non-gravitational stressor during spaceflight. While microgravity represents a chronic background challenge, the radiation environment of deep space introduces acute and stochastic risks. Beyond the continuous exposure to Galactic Cosmic Rays (GCR), astronauts are vulnerable to unpredictable Solar Particle Events (SPEs), characterized by sudden releases of energetic charged particles—predominantly protons—associated with solar flares and coronal mass ejections^8^. The energy spectrum of SPEs is distinct from that of GCR and is dominated by low-energy protons (<30 MeV). Although the aluminum hull of a spacecraft (~5 g/cm²) effectively attenuates these particles, the shielding provided by spacesuits during extravehicular activity (EVA) is substantially thinner (~0.5 g/cm²)^9^. As a result, during lunar EVA, these abundant low-energy protons are not fully stopped by the suit but instead deposit their energy within the astronaut’s body, primarily affecting superficial tissues such as the skin and the underlying epimysium of peripheral muscles.

This localized energy deposition creates a complex radiobiological scenario. Proton slowing is associated with dense ionization events and the generation of secondary radiation, including delta-ray electrons and characteristic X-rays. Consequently, tissues located just beyond the stopping range of the primary protons are not spared but are exposed to cascades of secondary particles and reactive chemical species generated by proton–tissue interactions.

Beyond canonical DNA damage, proton irradiation induces lipid peroxidation, disrupts membrane lipid organization, and alters plasma membrane rigidity, thereby affecting membrane-associated signaling complexes and ion channels^10^. Importantly, such effects may arise not only from direct particle traversal, but also from indirect mechanisms, including secondary radiation and oxidative stress generated in the surrounding medium, even when primary protons do not directly reach the cells. These membrane-level effects are increasingly recognized as critical determinants of radiation-induced cellular dysfunction.

Piezo1 is a mechanosensitive, non-selective cation channel that directly senses membrane tension and curvature, converting physical deformation into intracellular Ca²⁺ signals^11,12^. In skeletal muscle, Piezo1 contributes to myoblast fusion, satellite cell activation, and muscle regeneration, with Piezo1-mediated Ca²⁺ influx triggering downstream transcriptional and cytoskeletal remodeling pathways^13,14^. Importantly, Piezo1 gating is highly sensitive to the biophysical properties of the lipid bilayer—including membrane fluidity, stiffness, and lipid composition—rendering it vulnerable to processes that alter membrane mechanics independently of mechanical loading^15,16^.

The experimental design of the present study simulates this physiologically relevant SPE exposure scenario in vitro. We aimed to determine how proton radiation and the induced ionizing radiation affect the membrane structure of C2C12 cells and whether these alterations impair Piezo1 channel function, thereby providing a potential mechanistic link between radiation exposure and skeletal muscle dysfunction in space.

## Proton irradiation leads to an increase in the stiffness of the C2C12 cell membrane

**Fig. 1:**
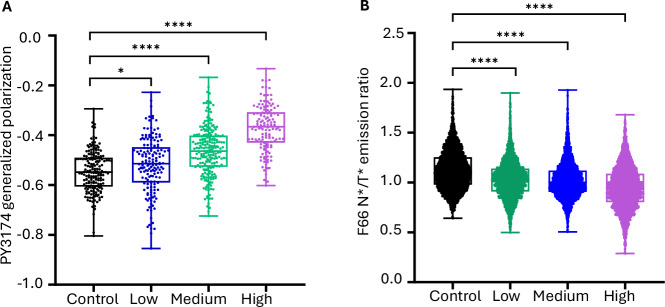
Effects of proton radiation on membrane biophysical properties. Control cells and those irradiated with various doses of proton radiation (Low: Φ = 2.5*10^9^ /cm^2^, Medium: Φ = 5*10^9^ /cm^2^, High: Φ = 1*10^10^ /cm^2^) were labeled with one of the two environment-sensitive fluorophores, the membrane hydration-sensitive PY3174 (**A**) or the dipole potential-sensitive F66 (**B**). Confocal microscopic images were taken at the midplane of cells in two wavelength ranges. During quantitative image analysis the plasma membrane pixels of individual cells were identified using a custom-written manually seeded watershed algorithm and the generalized polarization of PY3174 negatively correlating with the extent of membrane hydration or the N*/T* emission ratio of F66 negatively correlating with the magnitude of dipole potential was determined exclusively in pixels corresponding to the plasma membrane for each cell. Box plots with individual points were generated from median PY3174 generalized polarization or F66 N*/T* emission ratio values of at least 200 individual cells obtained in three independent experiments, which also display median values with quartiles. Asterisks indicate significant differences between the treated samples and the control (*p < 0.05, ****p < 0.0001, ANOVA followed by Dunnett’s test).

To determine whether proton irradiation alters plasma membrane structure through oxidative stress–related mechanisms, we quantified changes in membrane hydration and dipole potential, two molecular order-associated parameters reflecting distinct depths of membrane organization^17^. Membrane hydration was assessed by measuring the generalized polarization (GP) of the polarity-sensitive probe PY3174, which inversely correlates with water penetration into the lipid bilayer, while dipole potential was evaluated using the N*/T* fluorescence emission ratio of the voltage-sensitive dye F66. Using confocal microscopy and quantitative image analysis, we observed a dose-dependent increase in PY3174 GP values following irradiation, indicating reduced water penetration into the inner membrane layers (Fig. 1A). Representative images corresponding to these measurements are provided in Supplementary Fig. 1. In parallel, proton exposure induced a dose-dependent decrease in the F66 N*/T* ratio, consistent with an increased membrane dipole potential (Fig. 1B). One-way ANOVA revealed a significant effect of irradiation on PY3174 values (*F*(3,705) = 97.83, *p* < 0.0001). A similarly strong effect was observed for F66 measurements (*F*(3,15040) = 477.7, *p* < 0.0001). Detailed statistical parameters are provided in Supplementary Table 1. Together, decreased membrane hydration and elevated dipole potential indicate increased molecular order, demonstrating that proton radiation enhances membrane rigidity across both interfacial and deeper hydrophobic regions of the plasma membrane.

## Radiation exposure reduced the pharmacological activation of Piezo1 channels

**Fig. 2:**
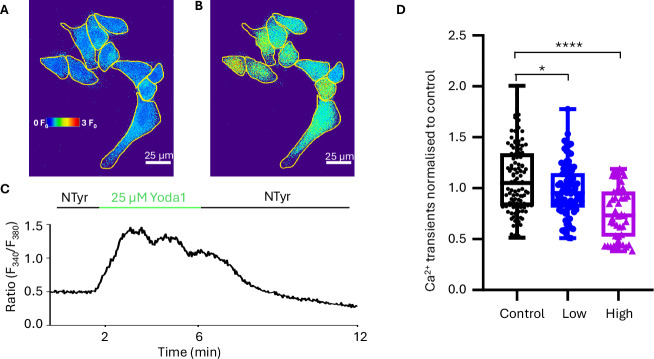
Calcium transients in C2C12 cells upon pharmacological activation of Piezo1 channels by Yoda1. Representative confocal images of Fura-2 AM-loaded C2C12 cells showing basal intracellular Ca²⁺ levels before stimulation (**A**) and increased Ca²⁺ signal following Yoda1 application (**B**). For functional measurements, cells were initially perfused with normal Tyrode’s solution (NTyr; 138 mM NaCl, 5.4 mM KCl, 0.5 mM MgCl_2_, 1.8 mM CaCl_2_, 11.18 mM HEPES, pH 7.4) for 2 min to establish baseline Ca²⁺ levels. Subsequently, 25 μM Yoda1 (prepared in NTyr) was applied by continuous perfusion for 4 min to ensure complete exchange of the chamber volume and attainment of the final concentration, thereby evoking maximal global Ca²⁺ transients. Yoda1 was not used as a pre-treatment. Finally, NTyr was reintroduced to wash out the agonist (6 min). Representative Ca²⁺ transient recorded from an individual cell is shown in (**C**). Amplitudes were quantified and normalized to the average response of non-irradiated control cells measured on the same experimental day (**D**). Proton irradiation resulted in a significant reduction in Ca²⁺ responses even at low fluence (Low: Φ = 2.5 × 10⁹ cm⁻²), with a further decrease observed at high dose (High: Φ = 1 × 10¹⁰ cm⁻²). Asterisks indicate statistically significant differences compared to control (*p < 0.05, ****p < 0.0001).

Following the detection of irradiation-induced changes in membrane rigidity, we examined the functional activity of Piezo1 channels. Experiments were conducted using the lowest and highest proton fluences. Intracellular Ca²⁺ levels were monitored with the calcium-sensitive dye Fura-2 to assess Piezo1 activation in response to the agonist Yoda1 (a representative record is shown on Fig. 2C). Basal intracellular Ca²⁺ concentrations did not differ between control and irradiated cells. In contrast, Yoda1-evoked Ca²⁺ transients were significantly reduced following proton irradiation. Panel D of Fig. 2 displays the distribution of Ca²⁺ transient amplitudes determined under specific conditions, normalized to control measurements performed on the same day, in which we observed a significant decrease at both proton fluence values (control: 1.085 ± 0.032, low: 0.979 ± 0.029, high: 0.765 ± 0.039). This reduction is unlikely to result from altered Piezo1 expression levels, as Western blot analysis did not reveal significant differences between control and irradiated samples (Supplementary Fig. 2).

Our findings demonstrate that proton radiation, simulating a solar particle event, induces dose-dependent changes in plasma membrane biophysical properties and mechanosensitive channel function in C2C12 myogenic cells. Specifically, proton exposure increased membrane rigidity, evidenced by reduced hydration and elevated dipole potential, and concurrently reduced the pharmacological activatability of Piezo1 channels. These results suggest that ionizing radiation can perturb membrane order, potentially compromising mechanotransduction independently of mechanical unloading. Such membrane-level damage has been recognized as a target of ionizing radiation in diverse cell types, where oxidative processes and lipid peroxidation alter membrane structure, fluidity, and associated signaling complexes (e.g., formation of lipid peroxidation products and perturbation of membrane polarity) in irradiated cells (e.g., CHO-K1 and lymphoblastic lines) and model systems^18^. The biological effects of ionizing radiation are generally attributed to both direct ionization events and indirect effects mediated by reactive species generated through water radiolysis. Although these processes were not directly assessed in the present study, radiation-induced oxidative stress is known to affect membrane organization, lipid packing, and dipole potential^10^.

The observed impairment of Piezo1 channel-mediated Ca²⁺ responses may have functional consequences for skeletal muscle maintenance and regeneration. Piezo1 is a bona fide mechanosensitive cation channel that transduces membrane tension into Ca²⁺ signaling and has been implicated in regulating myoblast fusion, satellite cell activation, and regenerative processes in skeletal muscle. Pharmacological activation of Piezo1 enhances myogenic precursor differentiation, and genetic ablation delays muscle regeneration following injury, underscoring its essential role in muscle physiology^19–21^. In this context, radiation-mediated alterations in membrane physical state may directly disrupt Piezo1 gating or its lipid microenvironment, thereby attenuating mechanosensitive signaling critical for muscle adaptation. Importantly, the combination of microgravity-induced unloading and radiation-induced membrane dysfunction may synergistically exacerbate muscle atrophy and regenerative deficits encountered during extended space missions. These insights point to membrane preservation and mechanosensitive channel integrity as potential targets for countermeasures aimed at mitigating muscle dysfunction in spaceflight.

## Methods

### Cell culturing

Immortalized, mouse C2C12 myoblast cell line was used. The culturing medium consisted of high-glucose DMEM (Biosera, Cholet, France, LM-D1108), 10% fetal bovine serum (Biosera, Cholet, France, FB-1200), 1% L-glutamine (Biosera, Cholet, France, XC-T1715) and 1% penicillin/streptomycin (Gibco by Life Technologies, Carlsbad, CA, USA, 15140122). Medium was changed every other day, and cells were subcultured at 80–90% confluence. Cultured cells were maintained in a proliferative, adherent state, and no differentiation protocol was applied. Conditions were verified using Invitrogen EVOS XL inverted microscope (Invitrogen, Carlsbad, CA, USA, 15339661) before the experiments were conducted. All experiments were performed on undifferentiated C2C12 myoblasts.

### Proton irradiation facility and beam characteristics

Meticulously cultivated cells were irradiated using protons in free air at the vertical beamline of the MGC-20 cyclotron located at the Institute for Nuclear Research (HUN-REN ATOMKI). The protons were accelerated by the cyclotron to a kinetic energy of 17 MeV. Following acceleration, the particle beam was transported to the dedicated irradiation site, where it was extracted from the high-vacuum environment into the atmosphere through a specialized 22 μm thick vacuum window foil composed of DURATHERM 600 stainless steel alloy.

To ensure the biological data collected was representative of the entire adherent cell population, quasi-homogeneous irradiation was required. This was achieved by collimating and defocusing the beam to produce a uniform 16 mm diameter beam spot. This spot size was calibrated to be centered within the 35 mm diameter polystyrene MatTek glass-bottom dishes used.

### Irradiation geometry and energy deposition

All irradiations were conducted with the Petri dish lids in place to maintain sterility and to serve as an initial scattering layer, mimicking the shielding of superficial layers. The proton beam experienced energy degradation as it traversed the vacuum window and the 0.51 mm thick polystyrene Petri dish lid. Calculations determined that upon reaching the surface of the liquid culture media, the kinetic energy of the protons was reduced to approximately 13 MeV.

As these protons passed through the culture medium, they deposited energy via ionization and excitation processes. Given the range of 13 MeV protons in water-equivalent material, the primary protons were expected to be fully stopped within the volume of the culture medium above the adherent cell layer. Consequently, the cells were not directly traversed by primary protons.

Under these conditions, the biological effects are likely mediated by indirect mechanisms, including (1) secondary radiation, such as delta-ray electrons and secondary photons generated within the medium, and (2) reactive species and radiolysis products formed in the irradiated medium, which may contribute to a radiotoxic microenvironment.

### Dosimetry and fluence control

Precise dosimetry was applied to simulate proton fluences relevant to Solar Particle Event (SPE) conditions. The exit window foil was maintained in galvanic contact with its metal holder, which was electrically insulated from the rest of the irradiation facility. This configuration enabled the foil holder to function as a high-sensitivity beam current monitor, while the irradiation unit containing the Petri dish served as a Faraday cup for charge integration.

The accumulated charge was carefully controlled within the range of 800–3200 pC ( ± 20%), depending on the specific bioassay. These charge values corresponded to proton fluences in the range of approximately Φ ≈ 2.5 × 10⁹ to 1 × 10¹⁰ cm⁻² at the target surface.

The selected fluence levels (Low: 2.5 × 10⁹ cm⁻², Medium: 5 × 10⁹ cm⁻², High: 1 × 10¹⁰ cm⁻²) were chosen to bracket the range expected during major Solar Particle Events (SPEs), as defined by Benton and Benton (2001), representing realistic exposure scenarios for astronauts during extravehicular activity (EVA) under current shielding conditions.

For membrane biophysical measurements, all three dose levels (low, medium, high) were applied to establish dose dependence. Based on these results, functional assays of Piezo1 activation were restricted to the low and high fluence conditions to capture the range of biological responses.

For Western blot experiments, a consistent charge corresponding to the high fluence condition (3200 pC; ~1 × 10¹⁰ cm⁻²) was applied.

### Western blot

Cells were lysed in buffer (20 mM Tris-HCl, 5 mM EGTA) supplemented with protease inhibitors. Samples were mixed with electrophoresis buffer, boiled at 95 °C for 5 min, and 30 μg total protein was separated on 10% SDS–polyacrylamide gels. Proteins were transferred to nitrocellulose membranes and blocked in 5% skim milk in PBS. Membranes were incubated overnight at 4 °C with primary antibodies against Piezo1 and GAPDH, followed by HRP-conjugated secondary antibodies for 1 h at room temperature. Signals were detected using enhanced chemiluminescence and imaged with a gel documentation system. Band intensities were quantified using ImageJ and normalized to GAPDH.

### Examination of membrane biophysical properties

For the investigation of membrane hydration, the generalized polarization of PY3174 (di-4-AN(F)EPPTEA; 4-[2-(6-Dibutylamino-5-fluoro-naphthalen-2-yl)-vinyl]-1-(3-triethylammonio-propyl)-pyridinium dibromide) (Potentiometric Probes, Farmington, CT), a Laurdan substitute with more favorable spectral properties, was quantified using confocal microscopy as described previously^22^. Control cells and those irradiated with various doses of proton radiation were labeled with 10 µM PY3174 for 20 min at room temperature, which was followed by acquiring images at the midplane of cells with an LSM880 confocal laser-scanning microscope (Carl Zeiss AG, Jena, Germany) after a 488-nm excitation and measuring the emission between 500 and 540 nm (I1) and 630 and 735 nm (I2). Images were analyzed in MATLAB (Mathworks, Natick, MA) by segmenting the images into membrane and nonmembrane pixels and identifying individual cells with a custom, manually seeded watershed algorithm. The median general polarization of PY3174 was calculated from the data of plasma membrane pixels for each individual cell after background subtraction using

1 $$\frac{{I}_{1}-{I}_{2}}{{I}_{1}+{I}_{2}}.$$

Changes in the magnitude of membrane dipole potential were quantified using confocal microscopy as in our previous studies^23^. Cells treated as above were stained for 20 min at room temperature with 10 nM F66 (N-[3-(40-dihexylamino-3-hydroxy-flavonyl-6-oxy)-propyl]N,N-dimethyl-N-(3-sulfopropyl)-ammonium, inner salt, a kind gift from Andrey Klymchenko (Université de Strasbourg, Strasbourg, France)) and images were acquired at the midplane of cells using an LSM880 confocal laser-scanning microscope. After an excitation at 405 nm, the emission was measured in two different wavelength ranges, 463–527 and 543–589 nm, corresponding to the normal (N*) and tautomeric (T*) forms of the excited state of the dye, respectively. Image analysis was carried out as described above with the difference that in these measurements the median N*/T* fluorescence emission ratio of F66 negatively correlating with the value of the dipole potential was calculated from the data of the identified plasma membrane pixels for each individual cell after background subtraction.

### Intracellular Ca^2+^-imaging

Cells were loaded with cell permeable, ratiometric, fluorescent Fura-2-AM calcium dye (Thermo Fisher Scientific, Waltham, MA, USA, F1201, 5 µM). Fura-2 was excited with a CoolLED pE-340fura light source mounted on a Zeiss Axiovert 200 M inverted fluorescence microscope. The excitation wavelengths were alternating between 340 and 380 nm, the emission was detected with a band-pass filter of 505–570 nm. The image acquisition and post processing were made with the ZEN Pro software. The calcium transients were calculated from the ratio of the images taken at 340 and 380 nm after background correction. The Piezo1 agonist Yoda1 (ProbeChem, Sanghai, China, PC-45542) was used for pharmacological activation. Yoda1 was applied at a final concentration of [25 µM]. For calcium imaging experiments, measurements were initiated in standard Tyrode’s solution (138 mM NaCl, 5.4 mM KCl, 0.5 mM MgCl2, 1.8 mM CaCl2, 11.18 mM HEPES, pH = 7.4; NTyr) (2 min), then cells were exposed to Yoda1 during recording, and Ca²⁺ responses were recorded under continuous presence of the compound (4 min). Once the effect had developed, the cells were perfused again with standard Tyrode’s solution (6 min).

### Statistical analysis

In the confocal microscopy experiments quantifying membrane biophysical parameters, measured data are represented on violin plots generated from data of at least 200 individual cells obtained in three independent experiments displaying median values with quartiles. One-way ANOVA was used for group comparisons. *F*-statistics, degrees of freedom, and *p* values are reported in the main text, with full statistical details provided in Supplementary Table 1. The *p* values were calculated by Dunnett’s test carried out after significant differences were obtained for between-group effects in ANOVA. Differences were considered significant when *p* < 0.05 (*p < 0.05, **p < 0.01, ***p < 0.001, ****p < 0.0001). For intracellular calcium measurements, unpaired t-test was applied to compare groups.

## Supplementary information

Supplementary Information

## Data Availability

The datasets generated and analyzed during the current study are not publicly available due to their complexity and because they are part of ongoing research but are available from the corresponding author upon reasonable request.
