# Supplementary material for: Proton radiation impairs mechanotransduction in C2C12 myoblasts
Source: NPJ Microgravity. 2026 May 14;12:67. doi: 10.1038/s41526-026-00609-w (PMC13421527; doi:10.1038/s41526-026-00609-w)
Supplement: Supplementary file 1 — Supplementary Information [file 41526_2026_609_MOESM1_ESM.pdf]

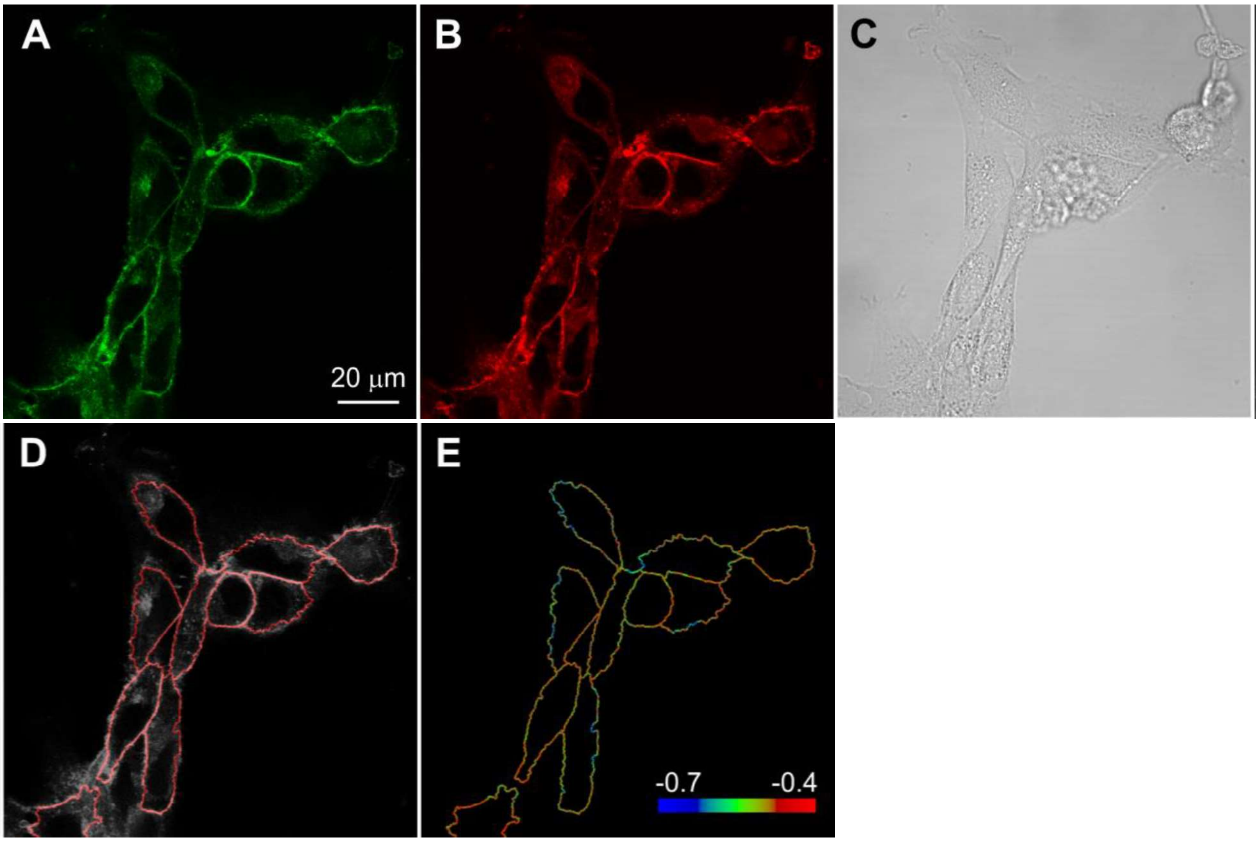

Supplementary Figure 1. Representative images showing image segmentation and analysis.

Cells irradiated by a low dose of proton radiation were labeled by PY3174 to measure hydration of the membrane. Images were recorded in two fluorescence channels corresponding to the blue (A) and red (B) part of the emission after excitation of the dye at 488 nm. A transmission image of the same microscope field is shown in panel C. Manually-seeded watershed segmentation was used for identifying the plasma membrane. The membrane mask shown in red is overlaid on the grayscale fluorescence image in panel D. The generalized polarization of the probe was calculated as described in the Methods section, and it is displayed on a color scale for membrane pixels only in E. The same image analysis algorithm was used for calculating the dipole potential-sensitive fluorescence emission ratio of F66.

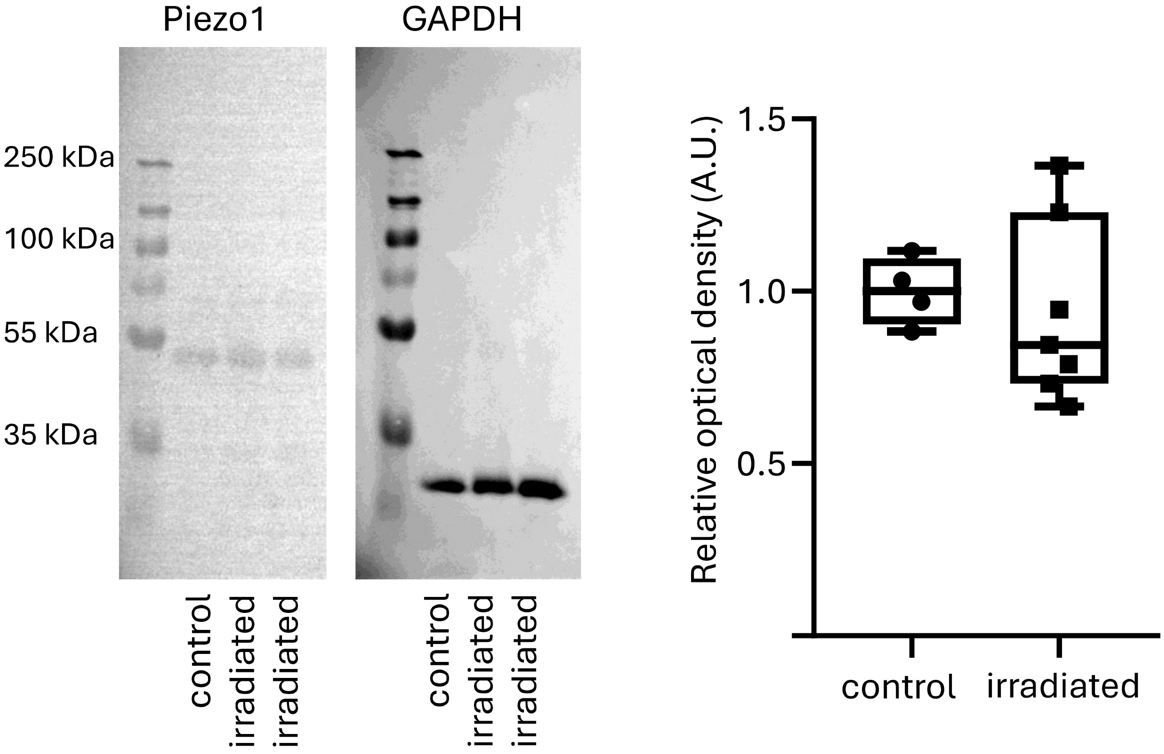

Supplementary Figure 2. Western blot analysis of Piezo1 expression in control and proton-irradiated C2C12 cells. (A) Representative immunoblot showing Piezo1 protein levels under the indicated conditions. (B) Densitometric analysis of Piezo1 expression normalized to GAPDH. No significant differences were observed between control and irradiated samples.

ANOVA table for PY3174 measurements

ANOVA table	SS	DF	MS	F	P value
Treatment (between columns)	2.716	3	0.9052	97.83	P<0.0001
Residual (within columns)	6.523	705	0.009253		
Total	9.239	708			

ANOVA table for F66 measurements

ANOVA table	SS	DF	MS	F	P value
Treatment (between columns)	51.69	3	17.23	477.7	P<0.0001
Residual (within columns)	542.5	15040	0.03607		
Total	594.2	15043			

Supplementary Table 1.

Summary of one-way ANOVA results for all datasets analyzed in this study. For each measurement, F-statistics, degrees of freedom, and exact p-values are reported.
